# Protection against Diarrhea Associated with *Giardia intestinalis* Is Lost with Multi-Nutrient Supplementation: A Study in Tanzanian Children

**DOI:** 10.1371/journal.pntd.0001158

**Published:** 2011-06-07

**Authors:** Jacobien Veenemans, Theo Mank, Maarten Ottenhof, Amrish Baidjoe, Erasto V. Mbugi, Ayse Y. Demir, Jos P. M. Wielders, Huub F. J. Savelkoul, Hans Verhoef

**Affiliations:** 1 Cell Biology and Immunology Group, Wageningen University, Wageningen, The Netherlands; 2 Department of Parasitology, Public Health Laboratory, Haarlem, The Netherlands; 3 Muhimbili University of Health and Allied Sciences, Dar es Salaam, Tanzania; 4 Laboratory for Clinical Chemistry and Haematology, Meander Medical Centre, Amersfoort, The Netherlands; 5 London School of Hygiene and Tropical Medicine, London, United Kingdom; New York University School of Medicine, United States of America

## Abstract

**Background:**

Asymptomatic carriage of *Giardia intestinalis* is highly prevalent among children in developing countries, and evidence regarding its role as a diarrhea-causing agent in these settings is controversial. Impaired linear growth and cognition have been associated with giardiasis, presumably mediated by malabsorption of nutrients. In a prospective cohort study, we aim to compare diarrhea rates in pre-school children with and without *Giardia* infection. Because the study was conducted in the context of an intervention trial assessing the effects of multi-nutrients on morbidity, we also assessed how supplementation influenced the relationship between *Giardia* and diarrhoea rates, and to what extent *Giardia* modifies the intervention effect on nutritional status.

**Methods and Findings:**

Data were collected in the context of a randomized placebo-controlled efficacy trial with 2×2 factorial design assessing the effects of zinc and/or multi-micronutrients on morbidity (n = 612; height-for-age z-score <−1.5 SD). Outcomes measures were episodes of diarrhea (any reported, or with ≥3 stools in the last 24 h) and fever without localizing signs, as detected with health-facility based surveillance. *Giardia* was detected in stool by enzyme-linked immunosorbent assay. Among children who did not receive multi-nutrients, asymptomatic *Giardia* infection at baseline was associated with a substantial reduction in the rate of diarrhea (HR 0.32; 0.15–0.66) and fever without localizing signs (HR 0.56; 0.36–0.87), whereas no such effect was observed among children who received multi-nutrients (p-values for interaction 0.03 for both outcomes). This interaction was independent of age, HAZ-scores and distance to the research dispensary. There was no evidence that *Giardia* modified the intervention effect on nutritional status.

**Conclusion:**

Although causality of the *Giardia*-associated reduction in morbidity cannot be established, multi-nutrient supplementation results in a loss of this protection and thus seems to influence the proliferation or virulence of *Giardia* or associated intestinal pathogens.

## Author Summary

*Giardia intestinalis* is a well-known cause of diarrhea in industrialized countries. In children in developing countries, asymptomatic infections are common and their role as cause of diarrhea has been questioned. In a cohort of rural Tanzanian pre-school children, we assessed the association between the presence of *Giardia* at baseline and subsequent diarrhea risk. The study was conducted in the context of a randomised trial assessing the effect of supplementation with zinc and other micro-nutrients on malaria, and half of the children daily received a multi-nutrient supplement. Surprisingly, we found that the presence of *Giardia* at baseline was associated with a substantial reduction in diarrhea risk. Multivariate statistical analysis showed that this protection could not be explained by differences in age or walking distance to the dispensary between children with and without *Giardia*. Because we cannot exclude that children differed in other (unmeasured) characteristics, we cannot draw firm conclusions about the causality of the observed association, but our findings support the view that the parasite is not an important cause of diarrhea in highly endemic settings. Striking was that the *Giardia*-associated protection was lost when children received multi-nutrients. Our data do not provide information about the mechanisms involved, but suggest that multi-nutrients may influence the compositionor pathogenicity of intestinal biota.

## Introduction

In developed countries, *Giardia intestinalis* (syn. *G. duodenalis*, *G. lamblia*) causes diarrhea while the prevalence of infections in the general population usually does not exceed 5% [1]. In developing countries, however, asymptomatic infections are much more common, with prevalence values in pediatric populations typically being around 30% [2]–[4], and reports on their association with diarrhea are inconsistent. Some reported an association with acute [5] and persistent [6] diarrhea, whereas several studies found no association [6]–[10], or even that *Giardia* infection was associated with protection against acute diarrhea [9], [11]–[14].

Because the role of *Giardia* as diarrhea-causing agent is controversial and re-infection can occur rapidly in developing areas where it is highly endemic, it has been recommended that children with asymptomatic infection should not be treated in such settings [15], [16]. This notion is challenged, however, by findings from surveys [17]–[21] and a prospective cohort study [22] suggesting that such infections may impair linear growth, presumably by reducing intake and causing malabsorption of nutrients. In addition, in a prospective cohort study, it was found that episodes of *Giardia* with diarrhea but not diarrhea itself were associated with impaired cognition, perhaps because infection can lead to deficiencies of zinc and other micronutrients that have been associated with deficits in cognitive development [23].

In the current study, we aim to compare rates of diarrhea in children with and without *Giardia* infection. Because the study was conducted in the context of an intervention trial that assessed the effect of multi-nutrient supplementation on malaria, we also assessed to what extent the relationship between *Giardia* and diarrhoea rates was influenced by supplementation. In addition, we explore whether the presence of *Giardia* infection at baseline modifies the response of nutritional indicators to multi-nutrient supplementation.

## Methods

### Study population

This study was part of a randomized placebo-controlled trial in children aged 6–60 months, with the primary aim to assess the effect of supplementation with zinc and other micronutrients on malaria rates (ClinicalTrials.gov, NCT00623857). It was conducted in a rural area in Handeni District, Northern Tanzania that is highly endemic for malaria. In a pilot survey among children aged 6–72 months in 2006 (n = 304), we found a high prevalence of *Giardia intestinalis* (30%; assessed by microscopic examination of a single stool sample per child), and only few cases of *Ascaris lumbricoides*, *Trichuris trichiura* or *Schistosoma intestinalis* (3%, 5% and 0%, respectively) (unpublished results). Residents in the area virtually all comprise poor farmer families engaged in subsistence farming, with oranges being produced seasonally as cash crops. Families are living in self-constructed clay houses, with very few having pit-latrines. Water for drinking and household use is collected from central shallow wells. Few people boil water before drinking. Access to health-care was limited until we constructed a research clinic at a central location in the study area, which provided free primary care to study participants.

### Design

Details about study design will be published elsewhere. In brief, between February and August 2008, we recruited all resident children aged 6–60 months, and excluded those with height-for-age z scores >−1.5SD, weight-for-age z-score <−3SD, haemoglobin concentration <70 g/L and with signs of severe or chronic disease, until attaining the target number (n = 600) (**Figure S1**).

The trial had a 2×2 factorial design with children receiving either multi-nutrients with zinc (**Table S1**), multi-nutrients without zinc, zinc alone (10 mg), or placebo. The levels of magnesium and vitamin C in the multi-nutrient supplement were below the upper limits that were based on osmotic diarrhea and related gastrointestinal disturbances as critical endpoints [24]. Supplements were color-coded and administered daily by community volunteers. Intervention groups were similar in baseline characteristics.

At baseline (on the day of enrollment), we collected venous blood in tubes suitable for trace element analyses (Becton-Dickinson, Franklin Lakes, NJ) and a fresh stool sample for each child in a vial that was pre-filled with sodium acetate-acetic acid-formalin (SAF) and stored in a refrigerator immediately after collection. A second vial with unfixed feces was stored in liquid nitrogen (−196°C) for subsequent genotyping. Whole blood hemoglobin concentrations were measured immediately using a portable photometer (Hemocue, Ängelholm, Sweden). An aliquot of plasma was stored in liquid nitrogen. A clinical officer recorded reported symptoms and performed a physical examination using standardised forms. We computed anthropometric indices as the average of two recordings, taken on consecutive days.

### Follow-up

We asked parents or guardians to bring their children to the research clinic if they noticed signs of illness. A clinical officer was on 24 h-duty and collected medical information on standardised forms that included a section on diarrhea.

A second survey, at 251 days (median; 95% reference range: 191–296 days) after enrolment, followed similar procedures as the baseline survey. Follow-up continued for all children until March 2009, when the study ended for all children simultaneously.

### Laboratory analyses

Stool samples were analyzed for the presence of *Giardia*-specific antigen by enzyme immunoassay (ProSpecT *Giardia* Microplate Assay, Oxoid, Basingstoke, UK). This test has a sensitivity and specificity of 93% and 99%, respectively, as compared to detection by microscopy in two sequential stool samples from individual subjects [25]. Plasma concentrations of ferritin, soluble transferrin receptor, folate and vitamin B12 were measured on a Beckman Coulter Unicel DxC880i system according to the manufacturer's instructions. Plasma concentrations of zinc and magnesium were determined by inductively-coupled plasma-mass spectrometry.

### Ethics Statement

The study was approved by ethical review committees in The Netherlands and Tanzania (National Health Research Ethics Review sub-Committee). We sought written individual informed consent; parents or primary caretakers were invited to sign (or thumbprint if illiterate) the informed consent form in the presence of a member of the community as impartial witness (who countersigned the form).

### Statistical analyses

Cases of diarrhea were defined as: a) all dispensary visits for parent- or guardian-reported loose or watery stools, with episodes being separated by at least 48 h of being without symptoms; or b) similar episodes with ≥3 loose or watery stools per 24-h period. Fever without localizing signs was defined as cases with reported fever that did not classify as malaria and were not accompanied by cough, diarrhea or other localizing signs. Thus cases of diarrhea and fever without localizing signs were mutually exclusive.

Data were analyzed using SPSS (v15·0 for Windows, SPSS, Chicago, IL, USA) and STATA (v11; College Station, Tx, USA). We report incidence rates and assessed group differences by Kaplan-Meier analysis with Tarone-ware test. Differences in the association between *Giardia* at baseline and morbidity outcomes between intervention groups were assessed by analysis within intervention strata, and directly by Cox regression analysis that included dummies for intervention groups and interaction terms. Cross-over between groups, whereby children who were initially infected became infection-free and vice versa in the course of the intervention period, may dilute potential effects of *Giardia* over time. For this reason, we restricted our primary analysis to first episodes, because an analysis of all events is probably more susceptible to such dilution of effect. However, because a substantial number of children experienced recurrent events and analysis of all events may better reflect total disease burden, we repeated these analyses based on all events, with robust estimates of the standard error to account for correlation between episodes within children. We explored potential confounding by adjusting for factors that were previously found to be prognostic for diarrhea and other morbidity outcomes (age, distance and height-for-age z scores). Children for whom *Giardia* infection status at baseline could not be determined were excluded from the analysis of the association between *Giardia* and disease rates.

Continuous outcome variables that were not normally distributed were log-transformed as appropriate. We used multivariate linear regression analysis with interaction terms to assess to what extent the effect of zinc and multi-nutrient supplementation (either alone or combined) on indicators of nutritional status depended on *Giardia* infection.

## Results

**Figure 1:**
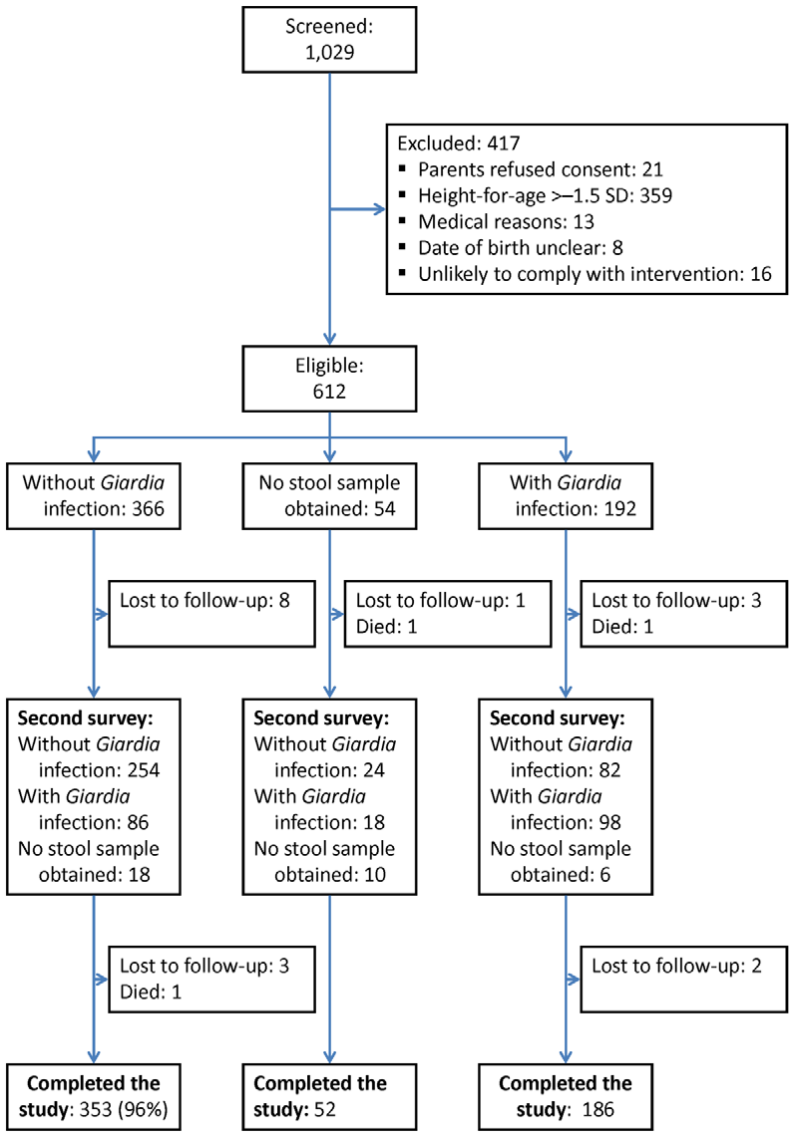
Study profile.

The study profile is shown in **Figure 1**. *G. intestinalis* was detected in 192 children (31%). We failed to obtain fresh stool samples for 54 children at baseline and for 50 children during the second survey, when 20 children (3%) were lost to follow-up (3 died; 2 were withdrawn by parents; 15 emigrated from the study area). Baseline characteristics are presented in **Table 1**. Children with *Giardia* infection were on average 3.3 months older than their uninfected peers, resided somewhat closer to the dispensary, had a lower prevalence of inflammation, marginally higher hemoglobin concentrations, as well as marginally lower plasma concentrations of soluble transferrin receptor and folate. All other biochemical indicators of nutritional status were similar, and we found no evidence that *Giardia* was associated with symptoms as reported by the mother (**Table S2**). The percentage of children who received antibiotic or anti-malarial treatment at baseline was similar in both groups.

**Table 1:** Baseline characteristics, by *Giardia intestinalis* infection status at baseline.

Characteristic	*Giardia*-positive	*Giardia*-negative	p
N	**192**	**366**	
Age, [mean]	35.0±14.5	31.7±16.1	0.02
6–17 mo	16% (31)	27% (97)	
18–35 mo	35% (68)	33% (122)	
36–60 mo	48% (93)	40% (147)	
Sex, boys∶girls (n∶n)	53%∶47% (102∶90)	46%∶54% (168∶198)	0.06
Distance between homestead and research dispensary, km	3.1 (1.8)	3.7 (2.3)	0.01
Mosquito net use	31% (117)	33% (59)	0.43
Anthropometric indices			
Height-for-age z-score, SD	−2.44±0.75	−2.40±0.67	0.49
Weight-for-height z-score, SD	−0.17±0.80	−0,07±0.83	0.20
*P. falciparum* antigenemia (n)^1^	44% (84)	43% (157)	0.46
Inflammation^2^	24% (46)	37% (134)	0.001
Biochemical indicators of nutritional status			
Hemoglobin concentration, g/L	104.8±12.1	101.7±12.6	0.005
Anemia^3^	65% (125)	71% (260)	0.09
Plasma zinc concentration, mmol/L^3^	9.0±2.4	9.0±2.3	0.86
Plasma ferritin concentration, µg/L^,^ 5	31.1 (26.8–36.1)	35.1 (32.0–40.4)	0.14
Iron deficiency^4^	20% (38)	17% (60)	0.17
Plasma sTfR concentration, mg/L^5^	2.4 (2.2–2.5)	2.6 (2.5–2.7)	0.02
Plasma cobalamin concentration, pmol/L^5^	347 (326–369)	333 (318–350)	0.33
Plasma folate concentration, nmol/L^5^	7.1 (6.5–7.7)	8.6 (7.9–9.2)	0.002

sTfR: soluble transferrin receptor. Values indicate mean ± SD unless indicated otherwise. For 54 children, infection status for *Giardia intestinalis* was unknown because fresh stool samples could not be obtained.

1 As determined by pLDH test; children with a positive test result were treated with artemether-lumefantrin, regardless of the presence or absence of malaria symptoms (see text);

2 Inflammation: whole blood C-reactive protein concentration >8 mg/L;

3 Anemia: hemoglobin concentration <110 g/L;

4 Iron deficiency: plasma ferritin concentration <12 µg/L (6 missing values);

5 Geometric mean (95% confidence interval).

At time of the second survey, 43% of children with *Giardia* infection at baseline no longer carried the parasite, while 23% of children who tested negative at baseline had become infected (Figure 1).

**Figure 2:**
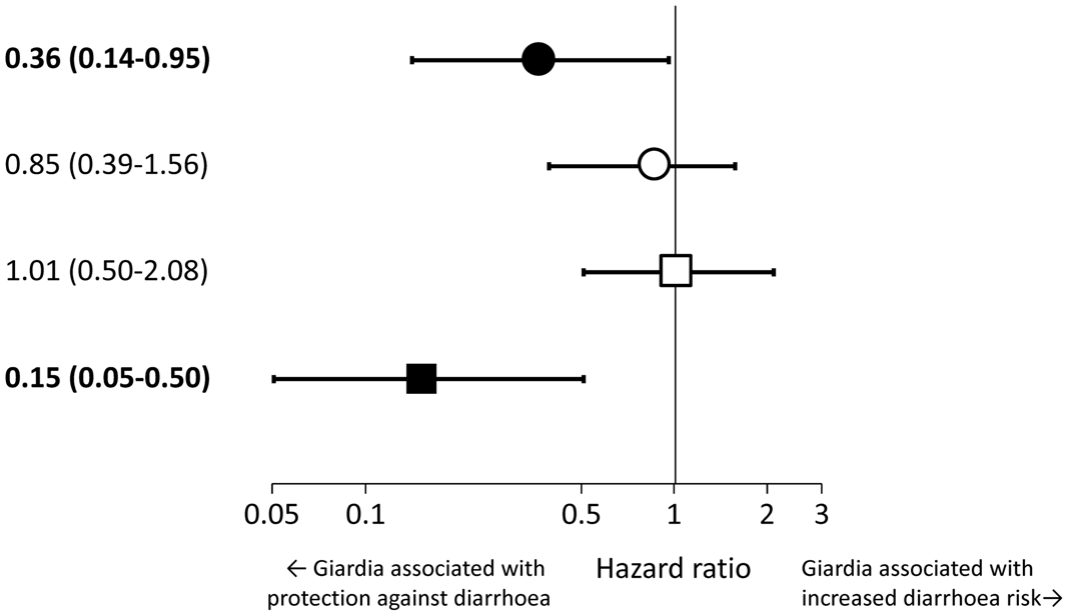
Association between *Giardia* and diarrhea rates, stratified by intervention. Estimates indicate crude incidence rate ratios (of first or only episodes with ≥3 watery stools/24 h), with 95% confidence intervals. An incidence rate ratio (IRR) of 1 (vertical line) indicates that *Giardia intestinalis* at baseline was not associated with diarrhea rates. An IRR of less than 1 indicates that *Giardia intestinalis* at baseline was associated with a reduction in diarrhea rates. Black dot: IRR in children receiving zinc. White dot: IRR in children receiving multi-nutrients without zinc. White square: IRR in children receiving multi-nutrients. Black square: IRR in children receiving placebo. *Giardia* was associated with protection against diarrhea among children receiving zinc or placebo, but not among children receiving multi-nutrients.

There were 3,268 clinic visits in a total follow-up time of 526 child-years. For 390 of these visits (12%), the parent or guardian reported diarrhoea, of which 223 episodes were accompanied by ≥3 loose stools in the past 24 h. Overall, the incidence of first episodes of diarrhoea was almost 50% lower among children with *Giardia* at baseline than among those without (**Table 2**). Similar effect estimates were obtained when including *all* episodes in the analysis (**Table 3**). When stratified by intervention group, the association between *Giardia* infection at baseline and diarrhea was similar in children receiving placebo as in those receiving zinc (in both groups the infection was associated with a protection). Likewise, this association was similar in both groups receiving multi-nutrients (no association in either group; **Figure 2**); we therefore combined children who received multi-nutrients with or without zinc (henceforth referred to as ‘with multi-nutrients’), as well as their peers who received zinc or placebo (‘without multi-nutrients’) as two separate groups.

**Table 2:** Incidence rates (*first* episodes) by *Giardia* infection status at baseline, and by intervention group.

	*Giardia*-positive (n = 192)		*Giardia*-negative (n = 366)		Incidence rate ratio [95%CI]		p
**Analysis of first episodes**							
Any reported diarrhea (223 episodes)^a^							
All	0.43	[58/135.6]	0.68	[147/217.6]	**0.63**	**[0.46–0.86]**	
Without multi-nutrients	0.29	[21/72.5]	0.72	[79/109.7]	**0.40**	**[0.24–0.66]**	
With multi-nutrients	0.58	[37/63.1]	0.63	[68/107.9]	**0.93**	**[0.60–1.41]**	*0.01*
Episodes with >3/24 h (157 episodes)^a^							
All	0.24	[36/149.3]	0.45	[109/244.7]	**0.54**	**[0.36–0.79]**	
Without multi-nutrients	0.11	[9/79.7]	0.46	[57/123.2]	**0.24**	**[0.11–0.50]**	
With multi-nutrients	0.39	[27/69.6]	0.43	[52/121.4]	**0.91**	**[0.55–1.47]**	*0.003*
Fever without localizing signs (172 episodes)^a^							
All	0.43	[49/144.8]	0.68	[110/255.2]	**0.78**	**[0.55–1.11]**	
Without multi-nutrients	0.31	[23/74.1]	0.48	[60/124.0]	**0.64**	**[0.37–1.05]**	
With multi-nutrients	0.37	[26/.70.6]	0.38	[50/131.2]	**0.97**	**[0.58–1.58]**	*0.25*

Values between brackets indicate the number of episodes/child-years observation time. Incidence rate ratios [95% CI] are crude estimates based on the analysis first or only events. An incidence rate ratio of <1 indicates that Giardia is associated with a reduced rate of diarrhoea.

Far right column: p-values for differences between incidence rate ratios in children with and without multi-nutrients (based on Cox proportional hazards model that included interaction term between multi-nutrients and *Giardia*).

Numbers in intervention groups: without multi-nutrients: n = 306 (96 Giardia positive, 185 negative, 25 missing); with multinutrients: n = 306 (96 Giardia positive, 181 negative, 29 missing).

a 19,12 and 13 cases of reported diarrhea, diarrhea with ≥3 loose stools/24 h and fever without localizing signs occured in children for whom *Giardia* status was unknown.

**Table 3:** Incidence rates (*all* episodes) by *Giardia* infection status at baseline, and by intervention group.

	*Giardia*-positive(n = 192)		*Giardia*-negative(n = 366)		Rate ratio[95%CI]		p
**Analysis of all episodes**							
Any reported diarrhea (390 episodes)^a^							
All	0.55	[95/172.1]	0.85	[266/311.9]	**0.66**	**[0.49–0.89]**	
Without multi-nutrients	0.35	[30/85.3]	0.88	[138/157.2]	**0.41**	**[0.25–0.67]**	
With multi-nutrients	0.75	[65/83.8]	0.82	[128/154.7]	**0.93**	**[0.63–1.36]**	*0.01*
Episodes with >3/24 h (223 episodes)^a^							
All	0.30	[51/172.1]	0.53	[164/311.9]	**0.58**	**[0.39–0.84]**	
Without multi-nutrients	0.16	[14/85.3]	0.52	[82/157.2]	**0.32**	**[0.15–0.66]**	
With multi-nutrients	0.43	[37/83.8]	0.53	[82/154.7]	**0.83**	**[0.53–1.30]**	*0.03*
Fever without localizing signs (232 episodes)^a^							
All	0.38	[66/172.1]	0.48	[150/311.9]	**0.79**	**[0.57–1.10]**	
Without multi-nutrients	0.32	[27/85.3]	0.57	[89/157.2]	**0.56**	**[0.36–0.87]**	
With multi-nutrients	0.45	[39/83.8]	0.39	[61/154.7]	**1.13**	**[0.71–1.80]**	*0.03*

Values between brackets indicate the number of episodes/child-years observation time. Rate ratios are crude estimates of the hazard rate ratios [95%CI] based on a Cox regression model that included multiple episodes per child. A rate ratio <1 indicates that *Giardia* is associated with a reduced rate of diarrhoea.

Far right column: p-values for differences between rate ratios in children with and without multi-nutrients (based on Cox proportional hazards model that included interaction term between multi-nutrients and *Giardia*).

Numbers in intervention groups: without multi-nutrients: n = 306 (96 *Giardia* positive, 185 negative, 25 missing); with multinutrients: n = 306 (96 *Giardia* positive, 181 negative, 29 missing).

a 19,12 and 13 cases of reported diarrhea, diarrhea with ≥3 loose stools/24 h and fever without localizing signs occured in children for whom *Giardia* status was unknown.

**Figure 3:**
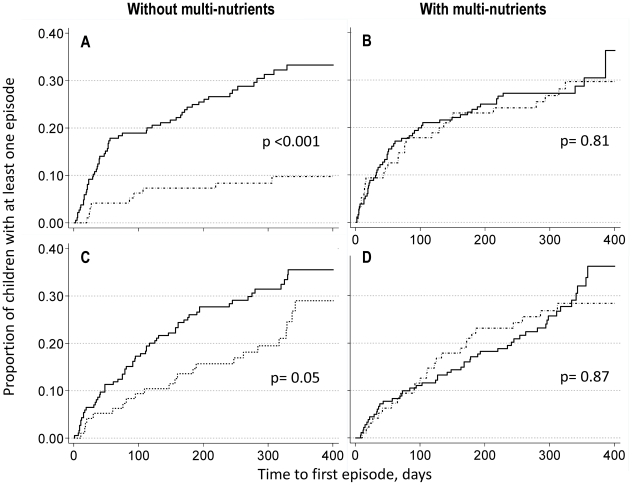
Kaplan-Meier curves for time to the first episode of diarrhea, or fever without localizing signs. Kaplan-Meier curves are shown for children without multi-nutrients (panel A and C) and for children with multi-nutrients (B and D). The upper two panels (A and B) show survival curves for diarrhea (with ≥3 watery stools/24 h: 157 cases). The lower two panels (C and D) show survival curves for fever without localizing signs (172 casdes). Solid lines: children without *Giardia intestinalis* infection at baseline. Dashed lines: children with *Giardia intestinalis* infection at baseline. p-values for group differences between children with and without *Giardia* (indicated in the panels) are obtained by Tarone-Ware test.

Thus analyzed, *Giardia* infection at baseline was associated with a substantial increase in time to first diarrhea episode with ≥3 watery stools/24 h (p<0.001), but only so among children without multi-nutrients, whereas no association was found between *Giardia* infection and diarrhea in those receiving multi-nutrients (**Figure 3**; top panels, and Tables 2 and 3).

**Figure 4:**
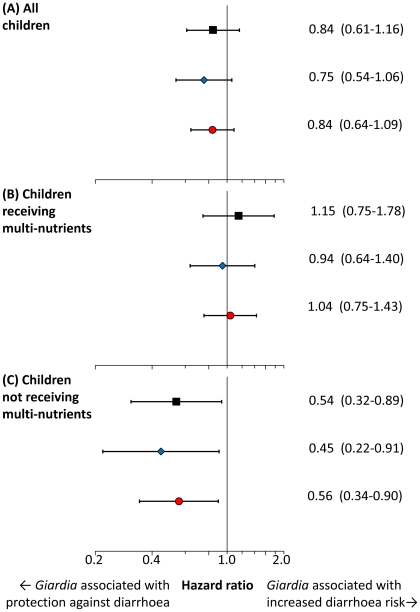
Adjusted estimates of the associations between *Giardia* and diarrhea, or fever without localizing signs. Hazard ratios (95%CIs) were calculated using multivariate Cox regression, adjusting for age (continuous), height-for-age z-score (continuous) and distance between homestead and research clinic (< or ≥4 km). Hazard ratios were assessed in all children (panel A), or for children with or without multi-nutrients separately (panel B and C). Further adjustment for sex, zinc deficiency, mosquito net use, weight-for-height z scores and inflammation at baseline led to virtually identical estimates (not shown). Black square: fever without localizing signs; blue diamond: reported diarrhea, ≥3 watery stools per day; red circle: reported diarrhea.

Adjustment for age and distance to the dispensary led to smaller but still substantial associations between baseline *Giardia* infection and diarrhea (all events), whilst interaction effects between infection and the multi-nutrient intervention remained virtually unchanged (**Figure 4**). Further adjustment for baseline factors previously found to be prognostic for diarrhea (height-for-age z-scores, sex, inflammation and use of mosquito nets) led to similar effect estimates (not shown). We also explored the association between *Giardia* infection and diarrhea within age classes in children without multi-nutrients; although the numbers of cases within these strata was low, all estimates pointed towards a protective association (HR: 0.36 [0.13 to 1.01], 0.81 [0.43 to 1.55], 0.19 [0.04 to 0.84] in children aged 6–17 months, 18–35 months and 36–60 months, respectively). Also, when restricting the analysis to children who were infected at both surveys (n = 98) and those who were never infected (n = 294), we observed very similar patterns and came to the same conclusions (not shown).

Similar patterns were seen for cases of fever without localizing signs: *Giardia* infection was associated with an increase in the time to first episodes of such fevers among those receiving zinc or placebo, but not among those receiving multi-nutrients. Adjusted estimates of hazard ratios (including all events) are shown in figure 3.

**Figure 5:**
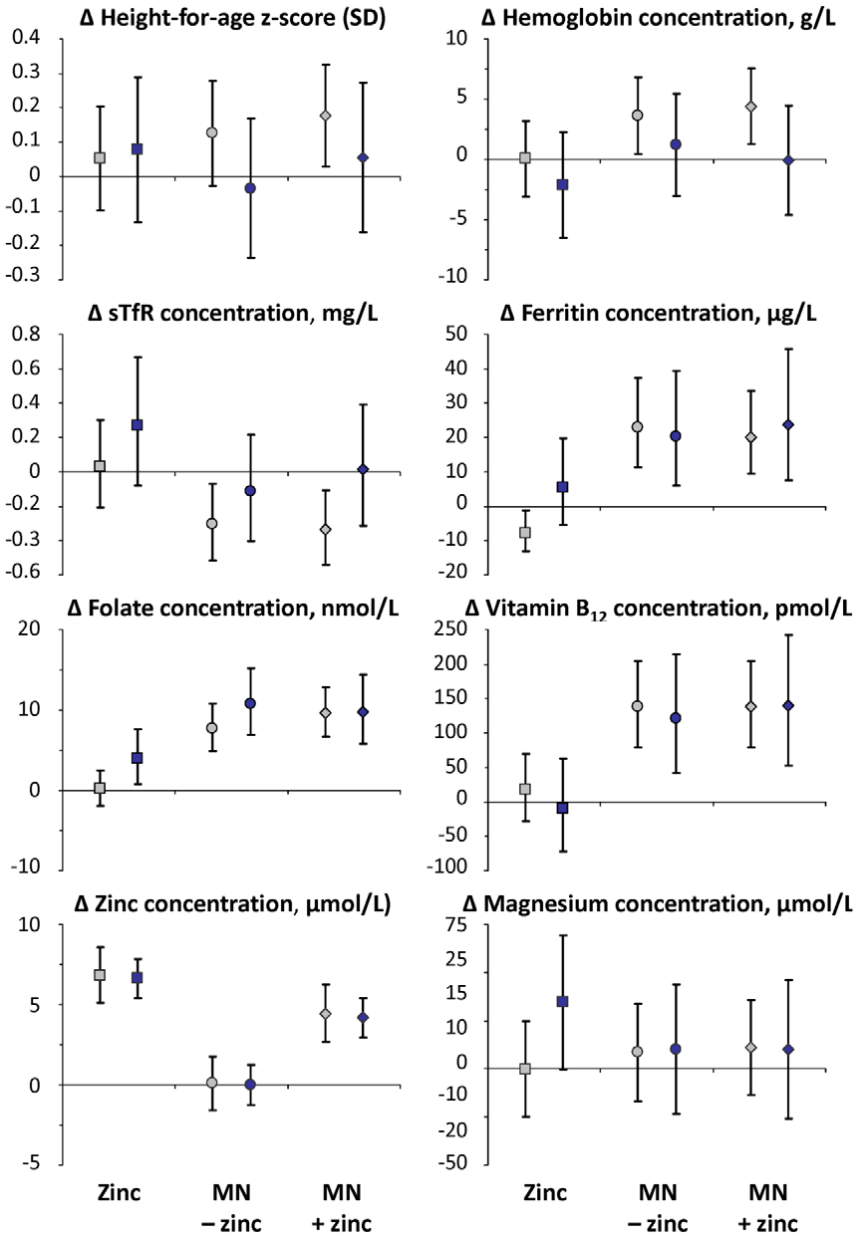
Effect of multi-nutrient supplementation on indicators of nutritional status, by *Giardia* infection status at baseline. sTfR: soluble transferrin receptor; MN: multi-nutrients. Effect estimates obtained for zinc, MN alone and MN plus zinc are indicated by squares, circles and diamonds respectively. Grey and blue: without and with *Giardia* infection, respectively. Dependent variables were log-transformed as appropriate, and expressed in natural units by exponentiation of estimates resulting from the analysis. For each nutritional status indicator investigated, effects are adjusted for the same indicator measured at baseline. Line bars indicate 95% CIs.

The effect of the multi-nutrients on height-for-age z-scores, hemoglobin concentrations and plasma transferrin receptor concentrations measured at the second survey tended to be greater in children without *Giardia* infection at baseline, whereas supplements seemed to have little effect in those who tested positive at baseline (**Figure 5**). The overall effects were rather small, and statistical evidence for differences in effect between children with and without *Giardia* was weak (p-values for interaction between *Giardia* and multi-nutrients: 0.13 [height-for-age z-scores], 0.24 [hemoglobin concentrations] and 0.32 [plasma soluble transferrin receptor concentrations]). Adjustment for age led to similar conclusions (not shown). For other indicators of nutritional status (plasma concentrations of zinc, magnesium, cobalamin, folate and ferritin), there was no evidence that *Giardia* infection influenced the effect of supplementation.

## Discussion

*Giardia intestinalis* infection at baseline was associated with a marked reduction in the rates of subsequent diarrhea among children receiving zinc or placebo, but not in those receiving multi-nutrients. Multi-nutrient supplementation among children with *Giardia* infection resulted in disease rates similar to those found in uninfected children. Similar patterns were observed for cases of fever without localizing signs.

Substantial cross-over occurred between groups in the course of the study, and this may lead to underestimates of differences between children with and without *Giardia* infection at baseline. Our Kaplan-Meier analysis indicates, however, that the protective association occurred almost from the start of the follow-up period, when presumably few cross-over cases had occurred.

Our study is limited by the observational nature of our data, which does not allow a conclusion that the protection observed was caused by *Giardia* infection. Although this association was still present after adjustment for age and other potentially confounding factors, we cannot exclude the possibility that children with *Giardia* infection differed from their uninfected peers in other unmeasured characteristics that are prognostic for diarrhea (e.g. sanitation, or previous or current exposure to other gastro-enteric pathogens [26]–[28] or health-care seeking behavior). We did not measure breastfeeding behavior, but it is unlikely that this could have explained the protective association found against diarrhea in children not receiving multi-nutrients: even in older children (aged 36–60 months), who are unlikely to be breastfed, *Giardia* infection was associated with a reduction in hazard rates by 81% (16% to 96%). Treatment with artemether at baseline (Table S2) may have had some effect on the prevalence or intensity of *Giardia* infections, which would argue against a causal role of the parasite in the observed protection [29].

Our findings support the view that the parasite is not an important cause of diarrhea in our study population. *G. intestinalis* comprises various genotypes, and its prevalence and its association with diarrheal symptoms seems to vary with geographic areas [30]. A recent study showed that *Giardia* infection was associated with protection against diarrhea, whereas *G. intestinalis* assemblage A was associated with acute diarrhea [13]. Thus, due to variation in genotypes and environmental factors, our findings may not apply to other populations, and further research is also needed to determine *G. intestinalis* genotype in this population.

It is not inconceivable that *Giardia* infection protects against diarrhea, for example by competing with or suppressing other enteric pathogens, or by inducing changes in mucosal immunity [e.g. 11], [31]. Chronic or repeated exposure to non-pathogenic *Giardia* genotypes may have induced immunity against more pathogenic genotypes. This cannot fully explain the protective effect observed, however, because the magnitude of the protective association found probably exceeds the *Giardia*-attributable fraction of diarrhea. *Giardia* infection may also be a marker of an unknown factor (e.g. previous exposure to other pathogens) that leads to protection against both diarrhea and fever without localizing signs.

Whatever the cause, *Giardia*-associated protection was no longer present when giving multi-nutrients. This interaction is supported by the magnitude of the differences between the subgroup effects, whilst the probability that the interaction is due to chance seems low. We believe it is highly unlikely that the estimates of the interaction effect are biased: because the intervention was randomly allocated it is improbable that an external factor (e.g. health care seeking behaviour) would coincidentally bias disease rates strongly towards a *Giardia*-associated protection in the zinc and placebo group, but not in both multi-nutrient groups.

Further studies are needed to evaluate how supplemental micronutrients influence the composition, proliferation and pathogenicity of intestinal biota, and the interaction of these biota with their host. Iron deserves special attention in view of findings that it can modify the profile of gut microbiota towards potentially more pathogenic [32], or enhance the virulence and invasion of *Salmonella enteritidis* [33], whilst a recent study suggests that supplementation with bovine lactoferrin, an iron binding-protein, reduced the prevalence of *Giardia* among in Peruvian preschool children [34]. A meta-analysis of intervention trials with iron showed a slightly increased risk of diarrhoea due to iron supplementation [35].

Our study findings do not support treatment of *Giardia* infections in symptom-free children, and question the benefit of providing multi-nutrient supplements in populations frequently exposed to diarrheal diseases.

In conclusion, *Giardia* infection at baseline was associated with a marked reduction in the rates of subsequent diarrhea. Our data suggest that it is a marker for the response in diarrhea to multi-nutrient supplements, that should be taken into the account when analysing trials assessing the effect of multi-nutrient supplementation on diarrhea.

## Supporting Information

Figure S1 **Participant flow.** 612 children were recruited between February and August 2008 (black line). A second survey was conducted between October 2008 and February 2009 (blue line). Follow-up stopped for all children simultaneously in March 2009 (dotted line).
(TIF)

Table S1 **Composition of the supplements.**
(DOC)

Table S2 **Symptoms reported at baseline, by** ***Giardia intestinalis*** **infection status at baseline.**
(DOC)

Checklist S1 **Strobe checklist.**
(DOC)
