# Supplementary figures and images for: Protection against Diarrhea Associated with Giardia intestinalis Is Lost with Multi-Nutrient Supplementation: A Study in Tanzanian Children
Source: PLoS Negl Trop Dis. 2011 Jun 7;5(6):e1158. doi: 10.1371/journal.pntd.0001158 (PMC3110167; doi:10.1371/journal.pntd.0001158)

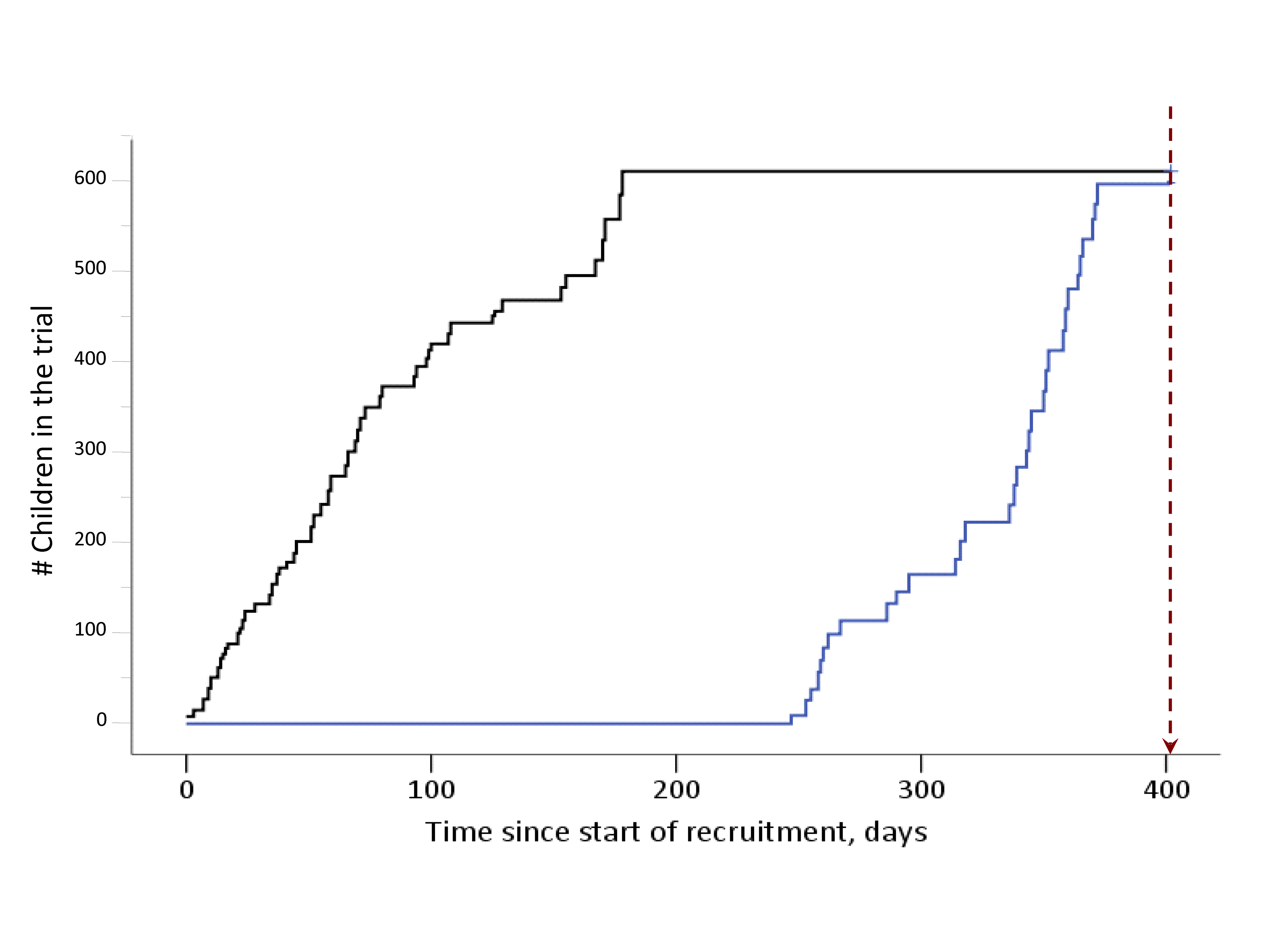

Supplement: Figure S1 — Participant flow. 612 children were recruited between February and August 2008 (black line). A second survey was conducted between October 2008 and February 2009 (blue line). Follow-up stopped for all children simultaneously in March 2009 (dotted line). (TIF) [file pntd.0001158.s001.tif]
