# Supplementary material for: Protection against Diarrhea Associated with Giardia intestinalis Is Lost with Multi-Nutrient Supplementation: A Study in Tanzanian Children
Source: PLoS Negl Trop Dis. 2011 Jun 7;5(6):e1158. doi: 10.1371/journal.pntd.0001158 (PMC3110167; doi:10.1371/journal.pntd.0001158)
Supplement: Table S1 — Composition of the supplements. (DOC) [file pntd.0001158.s002.doc]

**table S1.** Target dose and form of multi-nutrient supplement.

| Active substance |  | | Infants 6-12 months | | | Children 1-3 years | | Children 4-5 years | |
| --- | --- | --- | --- | --- | --- | --- | --- | --- | --- |
|  | Content | Form | RNI | UL | RNI | | UL | RNI | UL |
| Vitamin A | 450 µg RAE † | All-*trans* retinyl acetate (powder) | 400 µg | 600 µg | 400 µg | | 600 µg | 450 µg | 900 µg |
| Vitamin B_1_ | 0.625 mg | Thiamin mononitrate | 0.3 mg | ND | 0.5 mg | | ND | 0.6 mg | ND |
| Vitamin B_2_ | 0.55 mg | Riboflavin | 0.4 mg | ND | 0.5 mg | | ND | 0.6 mg | ND |
| Niacin | 6.6 mg | Niacine | 4 NE | ND | 6 NE | | 10 mg | 8 NE | 15 mg |
| Vitamin B_6_ | 0.575 mg | Pyridoxine | 0.3 mg | ND | 0.5 mg | | 30 mg | 0.6 mg | 40 mg |
| Folate | 93.75 µg ‡ | Folic acid | 80 µg | ND | 160 µg | | 300 µg DFE | 200 µg | 400 µg DFE |
| Vitamin B_12_ | 1.17 µg | Cyanocobalamid in mannitol | 0.5 µg | ND | 0.9 µg | | ND | 1.2 µg | ND |
| Vitamin C | 75 mg | Purified L-ascorbic acid | 30 mg | ND | 30 mg | | 400 mg | 30 mg | 650 mg |
| Vitamin D | 6.75 µg § | Vitamin D_3_ (cholecalciferol) | 5 µg | 25 µg | 5 µg | | 50 µg | 5 µg | 50 µg |
| Vitamin E | 6.6 mg | RRR-α-tocopherol acetate | 0.6 mg/kg bw ¶ | ND | 6 mg ¶ | | 200 mg | 7 mg ¶ | 300 mg |
| Vitamin K | 45 µg | Phylloquinone (vitamin K_1_) 5% | 10 µg | ND | 15 µg | | ND | 20 µg | ND |
| Zinc | 10 mg | Zinc as gluconate | 8.4 mg | 5 mg | 8.3 mg | | 7 mg | 9.6 mg | 12 mg |
| Iron | 18 mg | Ferrous fumarate | 18.6 mg | 40 mg | 11.6 mg | | 40 mg | 12.6 mg | 40 mg |
| Iodine | 90 µg | Potassium iodate | 90 µg | ND | 90 µg | | 200 µg | 90 µg | 300 µg |
| Copper | 340 µg | Cupric gluconate | 220 µg ¶ | ND | 340 µg ¶ | | 1 mg | 440 µg ¶ | 3 mg |
| Selenium | 20 µg | Sodium selenate | 10 µg | 60 µg | 17 µg | | 90 µg | 22 µg | 150 µg |
| Magnesium | 65 mg | Trimagnesium dicitrate anhydrous | 54 mg | ND | 60 mg | | 65 mg ║ | 76 mg | 110 mg ║ |

RNI: Recommended Nutrient Intake as established by WHO/FAO;^24^ UL: Tolerable Upper Intake Level as established by FNB/IOM; RAE: retinol activity equivalents; NE: niacin equivalents; DFE: dietary folate equivalents; TE: α-tocopherol equivalents; ND: Not derived

^†^ Equivalent to 1,500 IU

‡ Based on IOM estimates that 0.5 µg folic acid taken on an empty stomach corresponds to 1 µg DFE

§ Equivalent to 270 IU

¶ Values obtained from FNB/IOM (Dietary reference intakes for vitamin A, vitamin K, arsenic, boron, chromium, copper, iodine, iron, manganese, molybdenum, nickel, silicon, vanadium, and zinc. Washington DC: Institute of Medicine, 2001)

║UL applies to supplementary Mg
