# Supplementary material for: Protection against Diarrhea Associated with Giardia intestinalis Is Lost with Multi-Nutrient Supplementation: A Study in Tanzanian Children
Source: PLoS Negl Trop Dis. 2011 Jun 7;5(6):e1158. doi: 10.1371/journal.pntd.0001158 (PMC3110167; doi:10.1371/journal.pntd.0001158)
Supplement: Table S2 — Symptoms reported at baseline, by Giardia intestinalis infection status at baseline. (DOC) [file pntd.0001158.s003.doc]

**Table S2**. Symptoms reported at baseline, by *Giardia intestinalis* infection status at baseline.

| **Characteristic** | ***Giardia*-positive** | ***Giardia*-negative** | **p** |
| --- | --- | --- | --- |
| Symptoms reported at clinical assessment |  |  |  |
| Sick (according to mother) | 20% (39) | 23% (84) | 0.27 |
| Fever now or in the last 24 h | 16% (30) | 19% (68) | 0.43 |
| Sick according to clinical officer | 5% (10) | 6% (22) | 0.44 |
| Abdominal pain/discomfort | 24% (46) | 31% (112) | 0.06 |
| Diarrhea | 10% (19) | 12% (42) | 0.34 |
| Vomiting | 3% (5) | 4% (13) | 0.37 |
| Lack of appetite | 12% (23) | 11% (40) | 0.49 |
| Treatment provided at baseline ^1^ |  |  |  |
| Metronidazole | 2% (3) | 3% (10) | 0.29 |
| Mebendazole | 10% (19) | 8% (29) | 0.26 |
| Beta-lactam antibiotics | 12% (22) | 10% (36) | 0.32 |
| Artemether-lumefantrine | 44% (84) | 43% (157) | 0.46 |

^1^Children were treated on the day of enrollment.
